# 

**Published:** 1911-08-05

**Authors:** 


### THE WEEK'S NOVELTIES.

What after all is the best composition for a floor polish has not yet been decided, and therefore institutional workers should be interested to be informed of the latest scientific attempts at answering this question. Messrs. Martindale, of Liverpool, have sent us some samples of their "SANDRINGHAM" POLISH, which is described as sanitary and antiseptic. Our experiments seem to show that a case has been made out for its use, and we need add only that it is packed in 7 lb., 14 lb., and 28 lb. tins. Samples and prices can be obtained on application if accompanied by the mention of The Hospital.

### THE NEW PROFESSOR OF MEDICINE OF MANCHESTER UNIVERSITY.

Mr. Judson Sykes Bury, M.D. (London), B.Sc. (Manchester and London), Fellow of the Royal College of Physicians, London, has been appointed to the Chair of Clinical Medicine in the University of Manchester. Dr. Bury holds the position of physician to the Manchester Royal Infirmary, and was formerly Bradshaw Lecturer to the Royal College of Physicians. He has written an important work on Clinical Medicine, and is joint author with the late Dr. James Ross of a treatise on "Peripheral Neuritis."

### The Droitwich Waters.

A large party of members of the British Medical Association travelled by special train to Droitwich from Birmingham on Saturday last at the invitation of Viscount Cobham and Mr. Willis-Bund, the trustees of the Corbett Estate, to inspect the celebrated Brine Baths of this charming Worcestershire health resort. Many availed themselves of the opportunity afforded to bathe in the brine swimming bath, the water of which is so buoyant in consequence of the enormous quantity of brine held in solution that it is practically an impossibility to sink. This, to many, novel experience, was apparently very much appreciated, judging by the fact that at one time the bath contained between thirty and forty medical men. An excellent luncheon was provided in the Salters' Hall, after which the visitors proceeded to the delightful Brine Baths Park, where tea was served beneath the trees, the shade of which was very welcome in the almost torrid temperature. As our readers will doubtless recollect, a descriptive article on this Spa was contained in our issue of Feb. 25, 1911, when the treatment provided at Droitwich for disease of rheumatic and gouty origin was spoken of in the highest terms.

### INSTITUTIONAL LETTER-BOX.

### THE NATIONAL INSURANCE BILL.

*To the Editor of* The Hospital.

### THE QUESTION BOX.

### HOW IS A FOUNDATION STONE LAID?

*To the Editor of* The Hospital.

Sir,—The Committee are getting up a Cottage Hospital (at Skegness), the foundation stone of which is to be laid by the Princess Louise, and they have asked me if I have any information as to the ordinary procedure in these cases. I have kept no records, and I wondered whether you have a copy of your journal with an account of such a thing. If so, please forward, and I will meet the charge.—Yours faithfully,

Frank Tugwell.

[There is no fixed ceremony, but we are sending an account of an actual proceeding.—Ed. The Hospital.]

